# High Intensity Walking Reduces Frailty and Improves Physical Function Among Older Adults Living With HIV

**DOI:** 10.1093/geroni/igaa057.568

**Published:** 2020-12-16

**Authors:** Margaret Danilovich, Chad Achenbach, Chen Yeh, Lauren Balmert, Baiba Berzins

**Affiliations:** 1 CJE SeniorLife, Evanston, Illinois, United States; 2 Northwestern University, Chicago, Illinois, United States; 3 Northwestern University, Chicago, United States

## Abstract

Walking is a preferred mode of exercise for older adults, yet limited evidence exists on the optimal intensity to promote health gains, particularly among those with the frailty syndrome. Older adults aging with HIV have physical function impairments and higher prevalence of frailty, yet there is a paucity of evidence on therapeutic options to help these individuals maintain or improve their physical functioning. The purpose of this study was to investigate the feasibility and efficacy of a high intensity walking training (HIWT) intervention for pre-frail and frail older adults living with HIV. We enrolled n=11 older adults (>50 years of age and pre-frail or frail on the SHARE-FI). Participants underwent 16 walking sessions 2x/week consisting of 5 minute intervals of stair climbing, fast walking, weighted walking, balance tasks, and steps ups for a total of 30 minutes of high intensity (>70% of HRmax or >15 Rating of Perceived Exertion) activity. All participants were able to achieve the targeted high intensity levels throughout the sessions. We used a t-test to compare pre-post test means on a variety of physical performance measures. We found statistically significant improvements in the frailty score on the SHARE-FI, PROMIS fatigue, self-selected gait speed, Short Physical Performance Battery (SPPB), and Six Minute Walk Test. Importantly, all improvements were far above minimally clinically important differences suggesting this novel exercise approach may contribute to substantial improvements in physical function to reduce frailty in this population of older adults. Participants had no adverse events and were highly satisfied with training.

